# Safety Study of High-Speed Collisions between Trains and Live Intruder

**DOI:** 10.3390/s22228824

**Published:** 2022-11-15

**Authors:** Hai Zhang, Gengzhe Fu, Yongzhang Su, Yixin Yue, Wei Zhu, Chenyu Zhang, Yuxiang Lu

**Affiliations:** 1 Key Laboratory of Conveyance and Equipment of the Ministry of Education of China, East China Jiaotong University, Nanchang 330013, China; 2 CRRC Zhuzhou Locomotive Co., Ltd., Zhuzhou 412001, China; 3 The State Key Laboratory of Heavy Duty AC Drive Electric Locomotive Systems Integration, Zhuzhou 412001, China

**Keywords:** live intruder, passive train safety, finite element, cowcatcher

## Abstract

To investigate the safety of train collisions with live intruders under high-speed operation, a new 3D finite element laminated model of live intruder filling was constructed based on reconstruction using physical 3D scanning, with three outer layers of the model simulating the skin, three inner layers simulating bone, and internal filling simulating internal organs. The model was simulated in LS-DYNA with pendulum side collision, and the force–time and force–displacement curves of the collision between the pendulum and the living intruder were obtained, which were consistent with the curve trend of the results of the cadaver pendulum collision test by Viano in 1989, and the accuracy of the finite element model of the intruder was verified. Through the simulation calculation of high-speed collision between the train and two kinds of living intrusions, the maximum acceleration of the train body, the maximum lifting of the wheel pair, the deformation of the cowcatcher, and the maximum central load on the cowcatcher during the collision can be obtained. The results of the study show that at a collision speed of 110 km/h and different collision positions, the collision risk factor between the train and heavier organisms is relatively high, and the risk arising from frontal collisions is generally greater than that of offset collisions; despite this, all the indicators such as the maximum acceleration of the train, the maximum lift of the wheel pairs, the reduction in the length of the cowcatcher discharge per 5 m of space, and the maximum central load borne by the cowcatcher discharge are lower than the EN15227 standard. Additionally, the safety of the train is not affected and the components can work reliably.

## 1. Introduction

The safety of train operations has always been the top priority of railroad transportation. With the increasing speed of train operation, the probability of collision accidents is also gradually increasing. Although trains have a series of active safety measures, train service accidental collisions cannot be completely avoided; once a train collision accident occurs, the consequences are unimaginable [1]. Therefore, train collision safety technology has become a hotspot for research at home and abroad.

**Figure 1:**
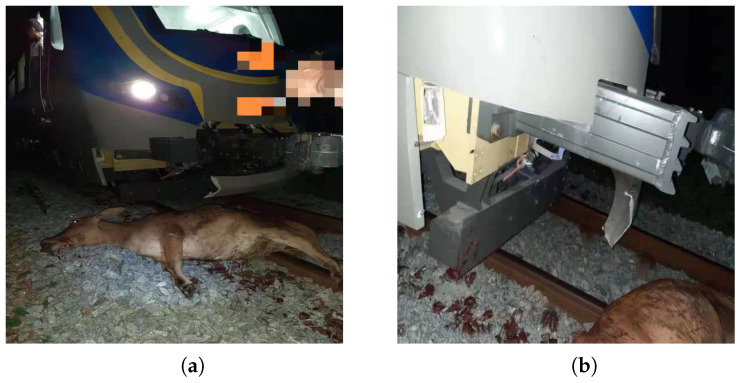
Collision between a train and a liveintruder in a Southeast Asian country: (**a**) Collision with a live intruder; (**b**) Train front-end damage.

From the information published by the Chinese State Railway Bureau, it can be found that train collisions are mainly divided into inter-train collisions, collisions between trains and buildings, and collisions between trains and intrusions, while train collisions are mostly dominated by the first two types of collisions. Therefore, domestic and foreign research also focuses on inter-train collisions and train–building collisions [2,3,4]. However, in recent years, with high-speed trains and high-speed rail lines being made available to improve the ability to travel, high-speed trains and live intrusion collisions have frequently occurred. Collisions with living bodies will cause serious railroad traffic casualties [5,6,7]. For example, on 2 June 2013, a G38 high-speed train from Hangzhou to Beijing was hit by a flying bird, resulting in a large crack in the front glass, and the train had to make an emergency stop at Nanjing South Station. Unlike China’s high-speed rail lines where railroad protection nets are installed, railroad lines in other countries around the world are generally less protected, and there is a possibility of collision between trains and live intruders in some areas [8]. Figure 1 shows the accident of a high-speed collision between an articulated rolling stock exported to a country in Southeast Asia and a live intruder. The outer cover of the front end of the train was broken, and the anti-crawler and excluder pushed the body of the intruder out of the track after the collision, thereby avoiding the danger of train derailment. This type of rolling stock belongs to the long formation group, the length of the driver’s cab is short, and it is impossible to arrange the regular energy-absorbing elements of the front end [9]. Therefore, it is necessary to conduct an extensive investigation on the safety of high-speed collision between trains and live intruders.

Collision simulation studies of trains generally precede crash tests [10]. Compared with collision tests, collision simulations are more economical and reproducible. Viano Huang et al. used actual cadaver experiments to derive biomechanical indices of the human thorax, abdomen, and pelvis and simplified the development of a finite element model of the human body during collisions [11,12]. Lu et al. used the multi-body dynamics method to analyze the collisions of different train formations at different speeds from linear and nonlinear perspectives and provided the recommended formula for calculating the train energy configuration [13,14]. Dias and Pereira used a multi-body dynamics approach to study the train collision problem and established 1D and 2D dynamics models to study the collision characteristics of trains and provide guidance for the pre-design of train crashworthy structures [15,16]. Koo et al. established a train collision multi-body model to study the phenomena of climbing, derailment, and lateral buckling of Korean high-speed trains under collision conditions and analyzed the influencing factors of derailment when the head car diagonally touches a rigid obstacle and a deformable obstacle [17,18]. Bingzhi Chen et al. used PAM-crash software to analyze the crashworthiness of the head car of high-speed rolling stock and studied the injuries caused by secondary collisions of passengers using a dummy model [19,20].

Based on the above findings, in order to realize the safety analysis of a high-speed collision between a train and live intruder, a new 3D finite element laminated model of a live intruder is constructed based on reconstruction with physical 3D scanning, and the pendulum side collision analysis is verified in LS-DYNA; the safety of train operation and the reliability of locomotive parts are discussed through the simulation calculation of high-speed collision between a train and live intruder.The contributions of the paper are as follows:

1. We analyze collisions between actual trains and living intruders, and consider various safety indexes such as the maximum acceleration, wheel pair lift, and maximum deformation of the cowcatcher during the high-speed collision, to judge the safety of trains and the reliability of locomotive parts during the driving process.
2. We propose a kind of organism model for collision (take a pig and cow as an example); based on the anatomy of an organism, we set the general position of each tissue of the organism and the corresponding material parameters, and simulate the side pendulum collision in LS-DYNA for comparison with the cadaver pendulum test. If the curve trend obtained by the model side pendulum collision is consistent with the test, the model will have a high accuracy rate. The model can be used not merely for studying train collisions but also in all kinds of live-body collision scenarios.
3. We verified the reliability of frontal and offset collisions of a pig and cow with trains.The collision simulation data are processed with strict reference to the standard EN15227 [21]. Therefore, our method can accurately determine whether the train meets the requirements of safe driving under live intruder collision conditions.

The remainder of this paper is arranged as follows: In Section 2, the finite element theory of collision simulation in relation to LS-DYNA and EN15227 collision response requirements is briefly introduced. In Section 3, the construction of a 3D model of the live intruder is presented and the validity of the model is demonstrated. In Section 4, the existing finite element model of the locomotive is presented. In Section 5, the calculation results are provided and each of the safety indexes during the collision are compared. Finally, our conclusions are provided in Section 6.

## 2. Preliminaries

### 2.1. Train Collision Theory

The train collision problem is a general contact and collision problem involving material nonlinearity, geometric nonlinearity, and contact interface nonlinearity [22,23].

The basic equations of collision dynamics can be expressed using equilibrium equations, geometric equations, physical equations in the *V* domain, and additional necessary boundary and initial conditions; the equations of motion of the system to be solved are obtained through a series of equivalent integral transformations as follow:

(1) $$M\ddot{a}\left(t\right)+C\dot{a}\left(t\right)+Ka\left(t\right)=Q\left(t\right)$$

where $\ddot{a}\left(t\right)$ denotes the nodal acceleration; $\dot{a}\left(t\right)$ represents the node velocity; a(t) represents the node coordinates; *M* represents the mass matrix; *C* indicates the damping matrix; *K* is the stiffness matrix; and *Q* stands for the nodal load vector.

The Lagrangian algorithm analysis of LS-DYNA is formed on the basis of Equation (1).

### 2.2. Crash Criteria Response

The crash calculation is based on standard EN15227, which states that the crashworthiness design of trains must meet the following requirements of EN15227:

1. No train crawling phenomenon occurs.
2. The average longitudinal deceleration in the survival space does not exceed 5 g.
3. When the collision condition simulated by the car group occurs, the survival space range of the people on board cannot be encroached.
4. Collision conditions to ensure that for each bogie at least one wheel pair does not break away from the rail so as to maintain contact with the rail.

## 3. Model Construction and Validation

### 3.1. Construction of a Three-Dimensional Model

The construction of a finite element model is the basis of finite element method research, and the construction of a finite element model of a live intruder requires geometric consistency with the actual living body shape and structure [24,25,26,27]. In the field of medical clinical diagnosis or biomedical engineering, the 3D reconstruction of living structures is often processed using CT (Computer Tomography) and MRI (Nuclear Magnetic Resonance Imaging) data [28,29,30]. The use of 3D solid scanning technology can quickly and accurately reconstruct the shape of the living body. Therefore, in this paper, based on the existing model and its anatomical structure, we use the ATOS optical scanner from GOM, Germany, to physically scan the live invasive element and obtain the point cloud data of the contour of the live intruder; we then import the point cloud data into Geomagic Studio software for the surface design and surface profile according to its structure to obtain CAD models of different parts of the animal body. This model will be used for the construction of the finite element model [31]. Table 1 shows the contour dimensions of the pig and cow.

#### 3.1.1. Point Cloud Data Acquisition and Processing

**Figure 2:**
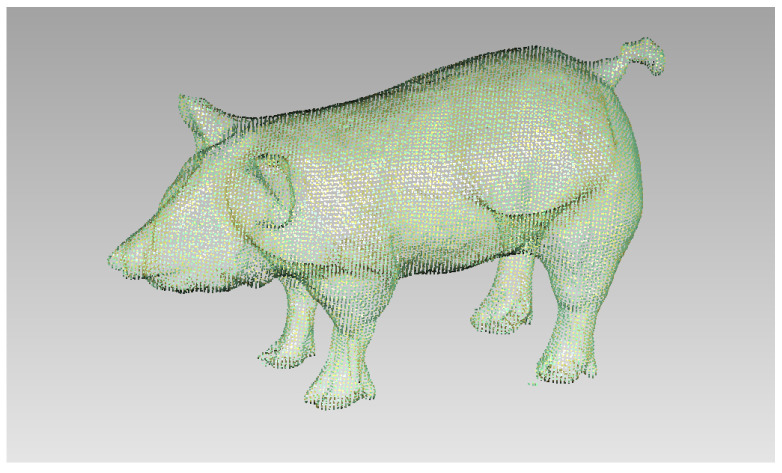
Point cloud data of pig profile.

**Figure 3:**
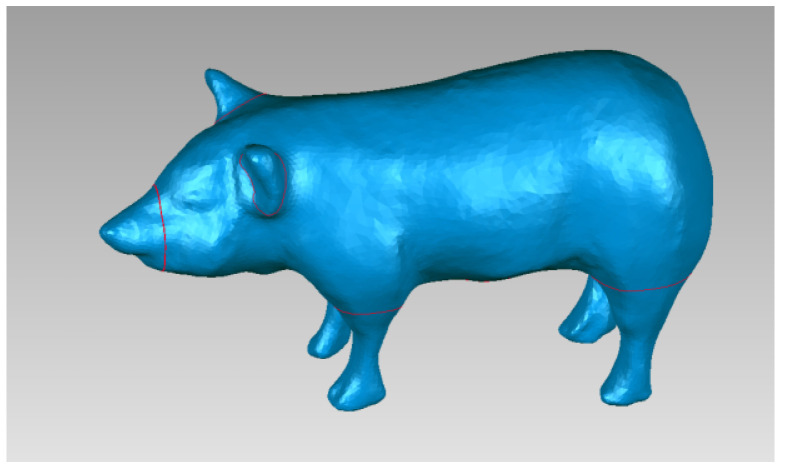
Pig polygon mesh model.

Taking the pig mode as an example, according to the existing standard animal model and its anatomical structure, the invasive element was physically scanned using an ATOS optical scanner from GOM, Germany, to obtain the point cloud data of the outline of the pig, as shown in Figure 2. After removing the unnecessary points, the model can be encapsulated with a polygon mesh. Figure 3 shows the encapsulated polygon mesh model of the pig.

#### 3.1.2. Model Segmentation and Surface Reconstruction

**Figure 4:**
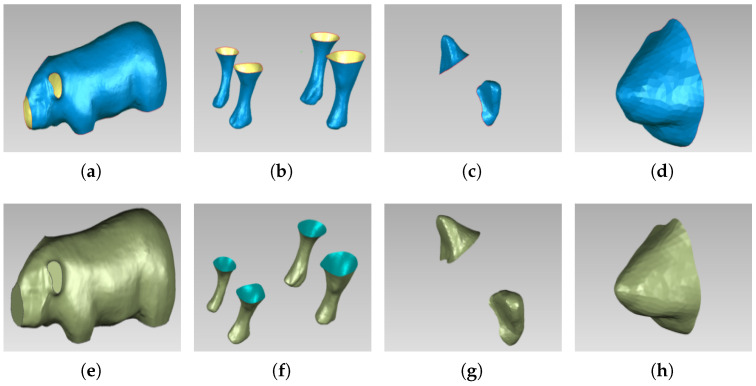
Polygon mesh model and surface model of different parts: (**a**) Polygon mesh model of pig body; (**b**) polygon mesh model of pig legs; (**c**) polygon mesh model of pig ears; (**d**) polygon mesh model of pig head; (**e**)surface model of pig ears; (**f**) surface model of pig legs; (**g**) surface model of pig ears; (**h**) surface model of pig head.

NURBS surfaces and boundary curves were generated based on the anatomy of the pig. Figure 4 shows the polygon mesh model and surface model of the body, ears, legs, and nose of the pig.

**Figure 5:**
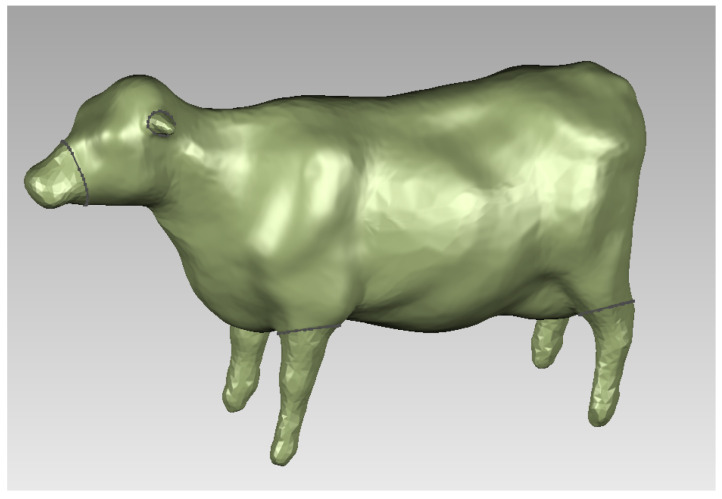
Surface model of cow.

Follow the same procedure as above for the cow model to obtain its surface model, as shown in Figure 5.

### 3.2. Construction of Finite Element Model

In terms of the anatomical structure of organisms, living intruders are composed of muscles, bones, organs, and other tissues. Organ-level and mass-block models are the two major types of organism models currently used, with the former being computationally intensive and the latter being too simple. In the study of high-speed collisions between trains and living intruders, on the one hand, the survival probability of living intruders is extremely low, and there is no need to examine the damage of living intruders; therefore, the organ-level modeling of live intruders is not required. On the other hand, to accurately simulate the collision energy absorption characteristics of living muscles and bones during collisions, it is not appropriate to use the simplified mass block model. Here, the filled 3D laminated finite element model is proposed to ensure the collision simulation resembles a real-world situation. Take the pig model as an example and divide the grid as follows.

#### 3.2.1. Element Mesh Division

**Figure 6:**
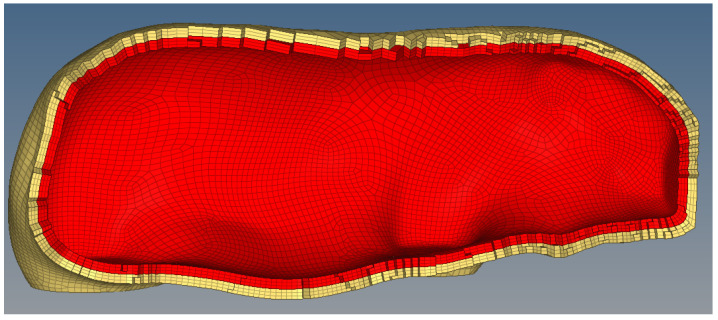
3D finite element laminated model of external laminated structure.

The 3D finite element laminated model of the external laminated structure is shown in Figure 6.

**Figure 7:**
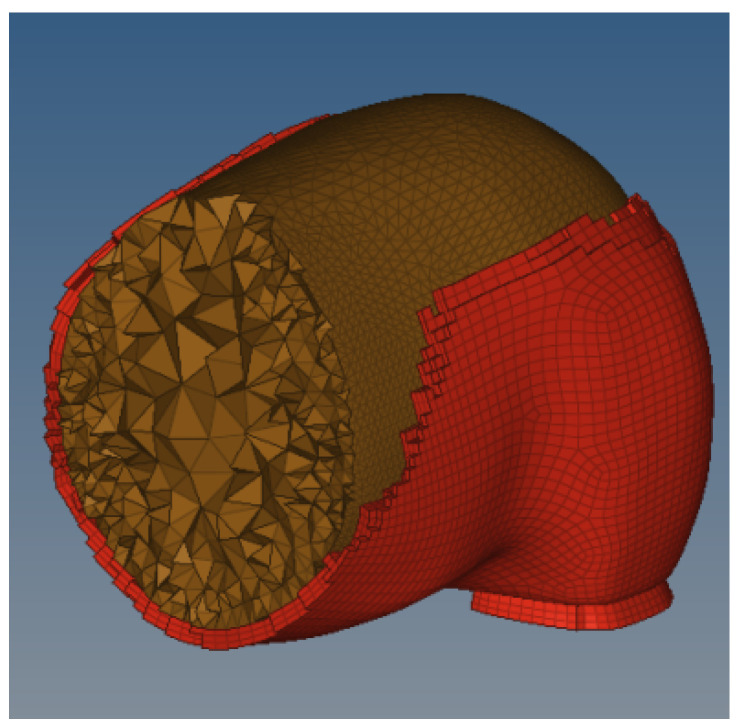
After filling (The brown part is the filling part).

The finite element model is composed of an external laminated structure in which the multi-layer tissue simulates the skin, bone, muscle, etc., of the organism, and the internal layer simulates various organs in the organism with mass points. After simulation tests, the organism model is prone to significant lateral compression and limb detachment when it collides with the train at a high speed, and the significant level of deformation has a large impact on the interaction force between the organism and the train. The large lateral compression is due to the large hollow in the 3D laminated model, and the limb shedding is due to the excessive stress on the joints of the model, which leads to the automatic deletion of the units and thus to limb shedding. In response to the above phenomena, the 3D laminated finite element model is supplemented with internal processing, as shown in Figure 7, and the simulation results of the improved finite element model are more accurate.

**Figure 8:**
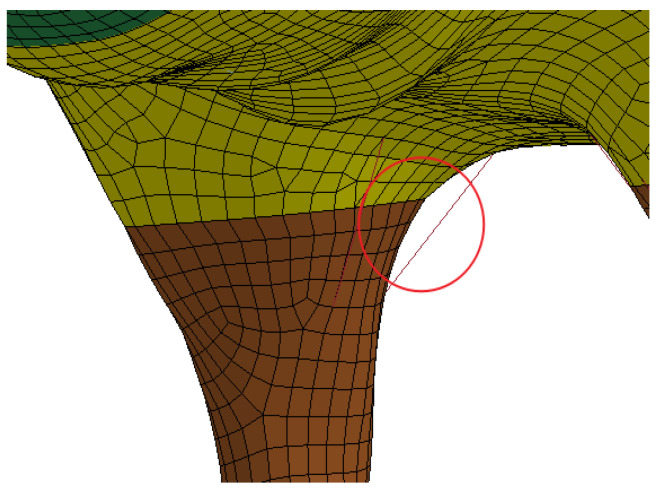
Ligamentous elements of living invader.

**Figure 9:**
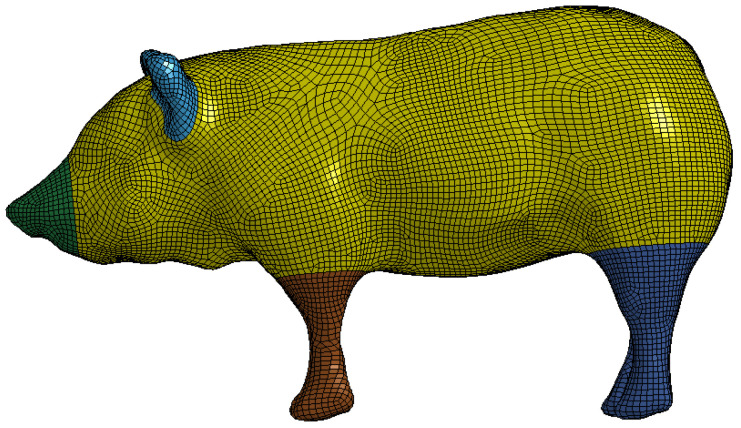
Front view of the overall pig model.

**Figure 10:**
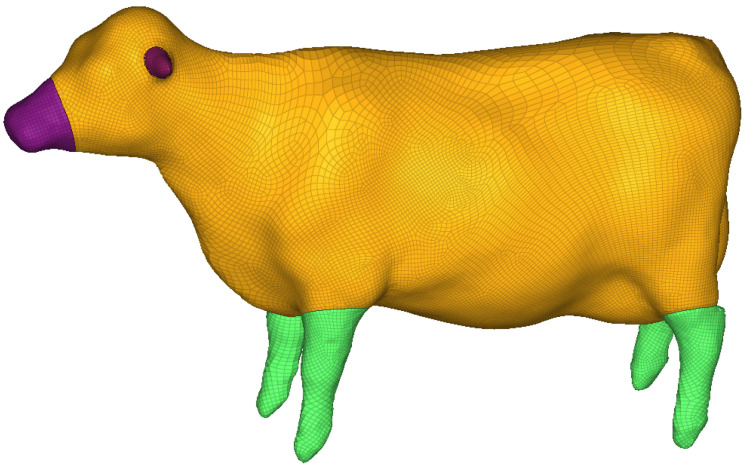
Front view of the overall cow model.

Referring to the anatomy of the organism, the joints are connected to the common nodes, as well as the ligaments simulated with beam elements to prevent the limbs from falling off, as shown in Figure 8. The overall finite element model is shown in Figure 9. The same method was used to obtain the bovine finite element model, as is shown in Figure 10.

#### 3.2.2. The Setting of Material Properties

By referring to the values of bone material parameters in the literature [32], the specific material parameters of bone, skin, and muscle are shown in Table 2.

The internal tissue material parameters are listed in Table 3.

The ligamentous tissue material parameters are shown in Table 4.

Bone damage is simulated by setting the failure plastic strain in the bone material, which ensures that the fracture of the rib is simulated by deleting the unit when the strain reaches its limit. In accordance with reference [33], the values of the mass weight of each main component are shown in Table 5.

#### 3.2.3. Define the Contact Type

In the collision environment, all parts of the model may be in contact; therefore, all models are included to define automatic face-to-face contact.

### 3.3. Simulation Verification

LS-DYNA software was used to analyze and verify the pendulum side impact simulation for the above-created live laminated model.

#### 3.3.1. Test Condition Setting

**Figure 11:**
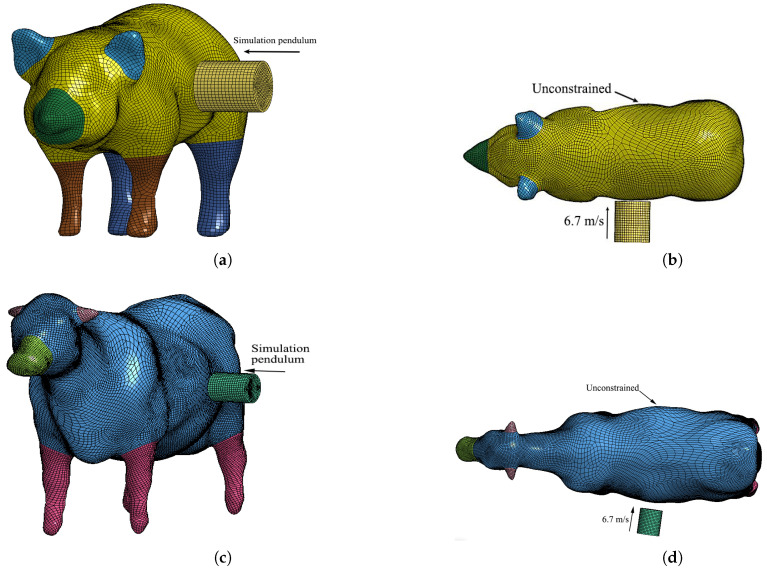
Simulation of pendulum side impact for laminated model: (**a**) Pig model side-impact model; (**b**) pig model simulation condition setting; (**c**) cow model side-impact model; (**d**) cow model simulation condition setting.

In Viano’s 1989 cadaver pendulum collision test mentioned in reference [25], in which the pendulum was set as a cylinder with a diameter of 150 mm and a weight of 23.4 kg, the initial collision speed was 6.7 m/s, and the total collision duration was 50 ms. The live body side impact simulation is shown in Figure 11.

#### 3.3.2. Collision Simulation Verification

**Figure 12:**
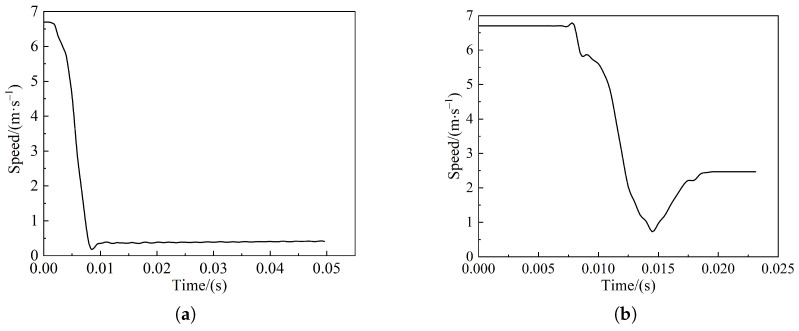
Pendulum speed–time curve during side collision: (**a**) Pig model pendulum curve; (**b**) cow model pendulum curve.

Figure 12 shows the pendulum velocity versus the time course curve when the pendulum impacts the side of the living body with an initial velocity of 6.7 m/s. In Figure 12a, the pendulum is blocked after contacting the live body, and the speed drops rapidly, reaching a minimum value of 9 ms; energy is absorbed through the deformation of the skin, muscles, and bones during the speed drop; after the velocity reaches the minimum value of 0.2 m/s, the energy generated by the pendulum is fully absorbed by the body, at which time the living body muscle and bone produce rebound and the velocity rises to about 0.5 m/s. The speed of the pendulum in Figure 12b drops to 0.7 m/s and then rises to 2.5 m/s with the same trend. The difference is related to the angle and position of the pendulum.

**Figure 13:**
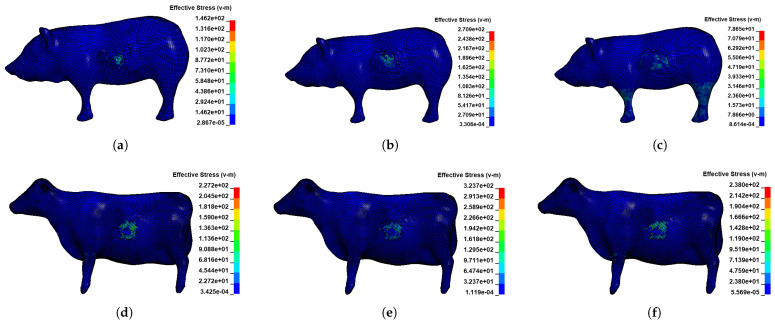
Stress clouds of the living body at each moment of the collision simulation: (**a**) Stress cloud at the moment before the maximum stress in the pig model; (**b**) stress cloud at maximum stress in the pig model; (**c**) stress cloud at the moment after the maximum stress in the pig model; (**d**) stress cloud at the moment before the maximum stress in the cow model; (**e**) stress cloud at maximum stress in the cow model; (**f**) stress cloud at the moment after the maximum stress in the cow model.

Figure 13 shows the stress clouds for some moments of the collision simulation. During the collision, large stresses appear in the muscle tissue.

**Figure 14:**
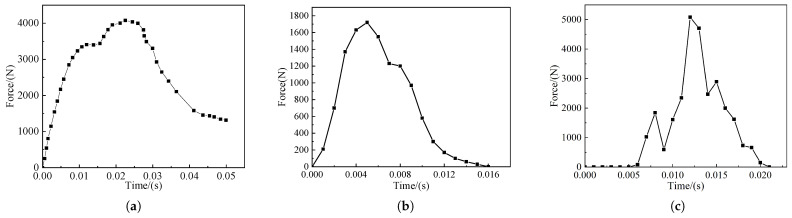
Force-time collision curves: (**a**) Force-time curve for cadaver testing; (**b**) pendulum simulation force–time curve of the pig model; (**c**) pendulum simulation force–time curve of the cow model.

**Figure 15:**
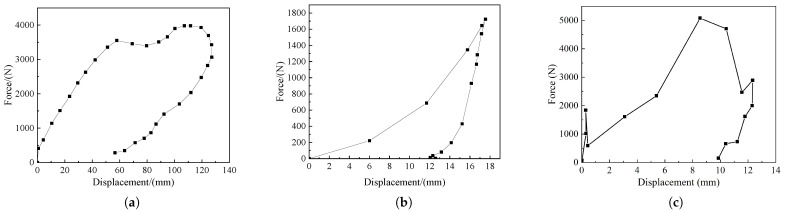
Force-displacement collision curves: (**a**) Force-displacement curve for cadaver testing; (**b**) pendulum simulation force–displacement curve of the pig model; (**c**) pendulum simulation force–displacement curve of the cow model.

Figure 14b,c show the live model impact force–time curve, Figure 15b,c show the live model impact force-displacement curve, and Figure 14a and Figure 15a show the impact force–time curve and impact force–displacement curve of the test results in the literature [25], respectively. From Figure 12 and Figure 13, it can be seen that the results of the live model calculations are in good agreement with both the human test results and simulation calculations in the reference [25], and the results indicate that the impact force reaches its maximum value at about 40% of the total calculation time; furthermore, the contact force continues to rise with the increase in displacement, while contact ends at 25% of the return displacement. The differences in the magnitudes of contact and impact forces observed in the literature are due to inconsistencies in the material parameters of the laminated model, and the deviations in the values are within the allowable range. The simulation results show that the 3D finite element laminated model of the live intruder can reduce the workload of finite element model building, reduce the calculation volume, save time and improve efficiency, and at the same time, it can accurately reflect the deformation of the animal body during the collision to meet the subsequent calculation requirements.

## 4. Finite Element Modeling of Train

**Figure 16:**
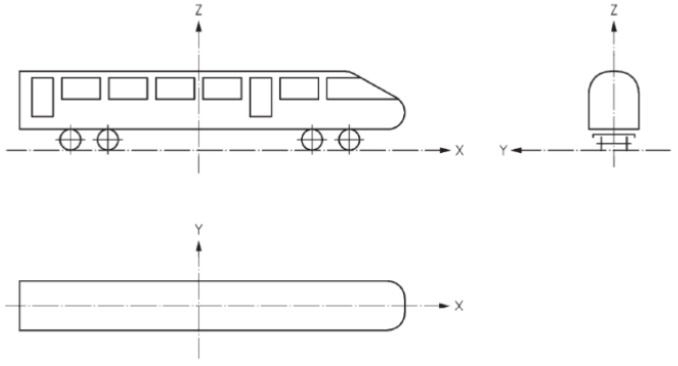
Train finite element model coordinate setting.

For finite element modeling, the following Cartesian coordinate system is used: the *x*-axis is positive along the longitudinal direction of the train and to the right, the *z*-axis is positive along the vertical direction of the train and upward, the *y*-axis is positive along the constant direction of the train, and the coordinate axes are arranged by the right-hand rule, as shown in Figure 16.

The training model consists of 6 cars, including 1 head car, 1 tail car, and 4 intermediate cars, and the weight of the equipment on the car is counterweighted by adding mass points. The total weight is 209.335 tons [34].

**Figure 17:**
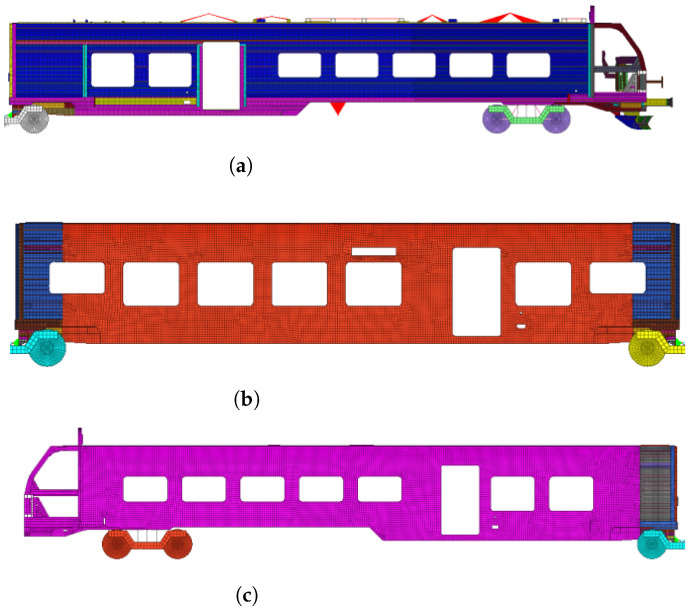
Finite element model of the train: (**a**) Finite element model of the head car; (**b**) finite element model of intermediate vehicle; (**c**) finite element model of the tail train.

Figure 17 shows the mesh model of the head car, tail car, and intermediate car of the train. The whole train contains 1,849,745 nodes and 2,019,890 elements, including 1,833,721 shell elements, 342,215 3D solid elements, 44 mass elements, 81 spring elements, 126 nodal rigid bodies, 64 beam elements, and 7 flexible connection elements. The typical material parameters of the finite element model of the train are shown in Table 6.

**Figure 18:**
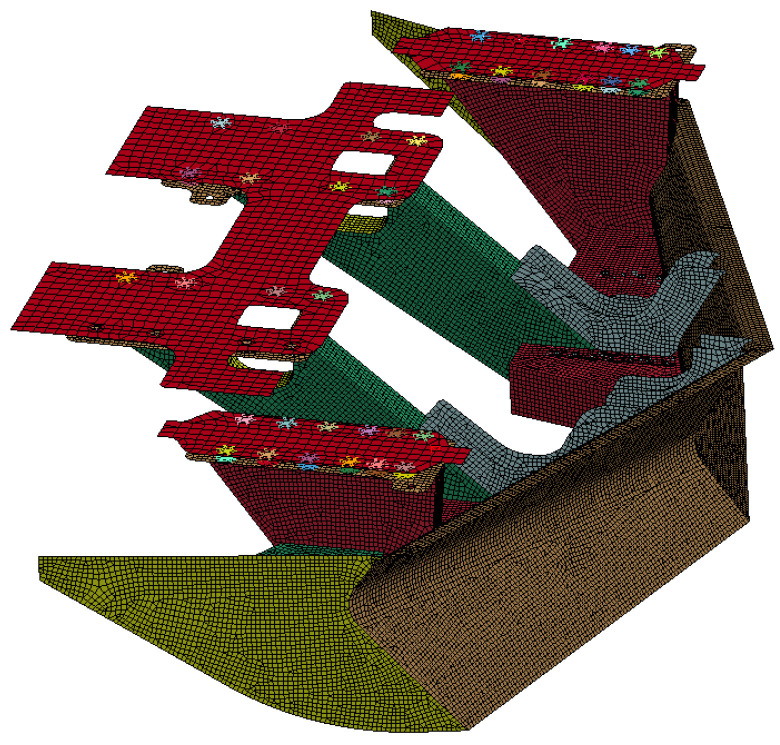
Finite element model of the cowcatcher.

The cowcatcher is modeled using shell elements, while the bolted connection to the front end is simulated using beam elements. This is shown in Figure 18.

## 5. Simulation Analysis

According to EN 15, 227 [21], trains are classified according to their crashworthy design. The four classifications of rail vehicles are indicated in Table 7 concerning the interrelationship between the application categories and the trains.

**Figure 19:**
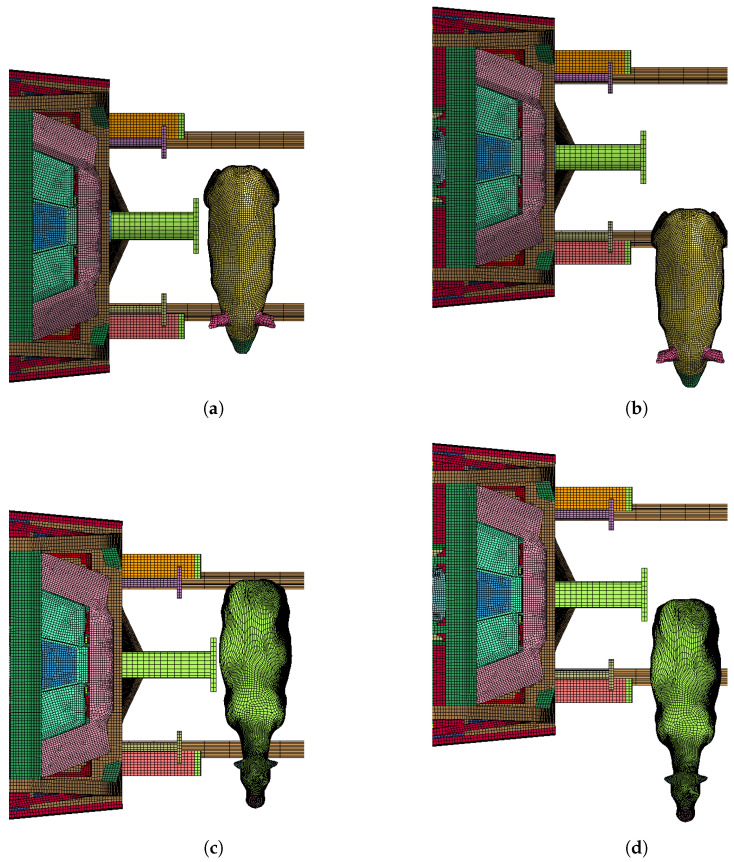
Collision Location: (**a**) Pig head-on collision; (**b**) Pig offset collision; (**c**) Cow head-on collision; (**d**) Cow offset collision.

**Figure 20:**
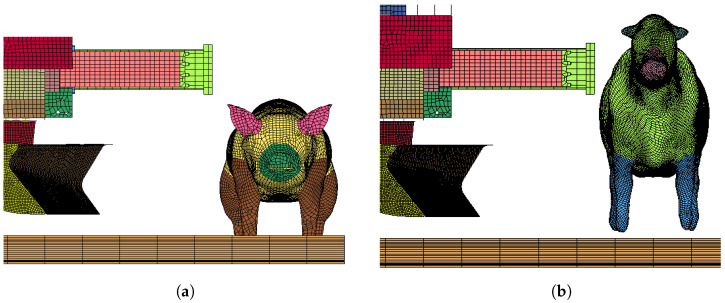
(**a**) Pig model; (**b**) Cow model.

According to the standard, different train collision-resistant design categories and different operating conditions to verify the collision resistance of vehicles have different expected collision scenarios; the object of this calculation is to evaluate the C-I category, that is, a train with a speed of 110km/h and a stationary live intruder on the track in front of the car, as shown in Table 8. In most cases, trains and intruders tend not to collide head-on; therefore, two possible cases of head-on and offset collisions are proposed in this paper to target different working condition requirements. Figure 19 shows the frontal collision and offset collision position settings for the pig and cow models. Figure 20 shows the front view of the initial setup of the collision.

**Figure 21:**
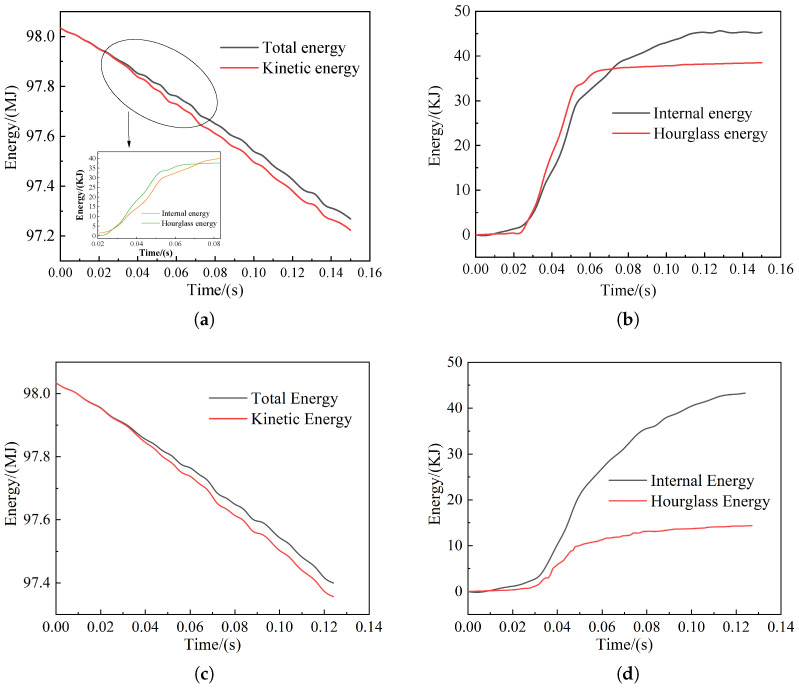
Collision energy curve: (**a**) Total energy and kinetic energy curve of frontal collision of pig; (**b**) internal energy and hourglass energy curve of frontal collision of pig; (**c**) total energy and kinetic energy curve for pig bias collision; (**d**) internal energy and hourglass energy curve for pig bias collision.

**Figure 22:**
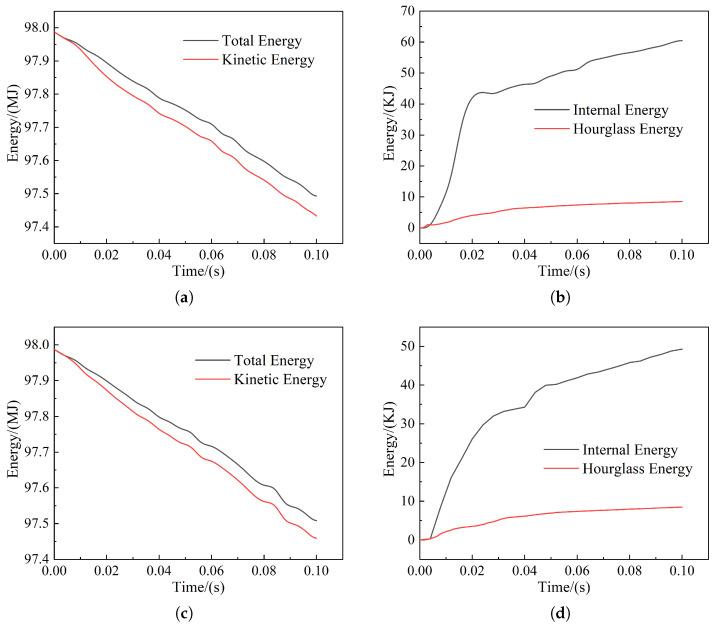
Collision energy curve: (**a**) Total energy and kinetic energy curve of frontal collision of cow; (**b**) internal energy and hourglass energy curve of frontal collision of cow; (**c**) total energy and kinetic energy curve for cow bias collisions; (**d**) internal energy and hourglass energy curve for cow bias collisions.

The curve of train energy with time is shown in Figure 21 and Figure 22. From the figures, it can be seen that the initial total energy of the model collision is maintained regardless of the frontal or offset collision. The initial kinetic energy difference between the car body in collision with the pig model and the cow model is small, for which the values are 98.03 MJ and 97.96 MJ, respectively. The internal energy and hourglass energy will change differently due to the different collision objects and collision locations. Because of the different weight of the live model, the internal energy generated by the collision involved in the heavy cow model is more than that of the light pig model, in which the internal energy of the bovine frontal collision changes the most, exceeding 60 KJ; the hourglass energy of the porcine frontal collision changes the most, reaching 40 KJ. Overall, the hourglass energy in all collisions is much lower than 5% of the total energy, meeting the requirements of the collision with high accuracy.

### 5.1. Train Acceleration

**Figure 23:**
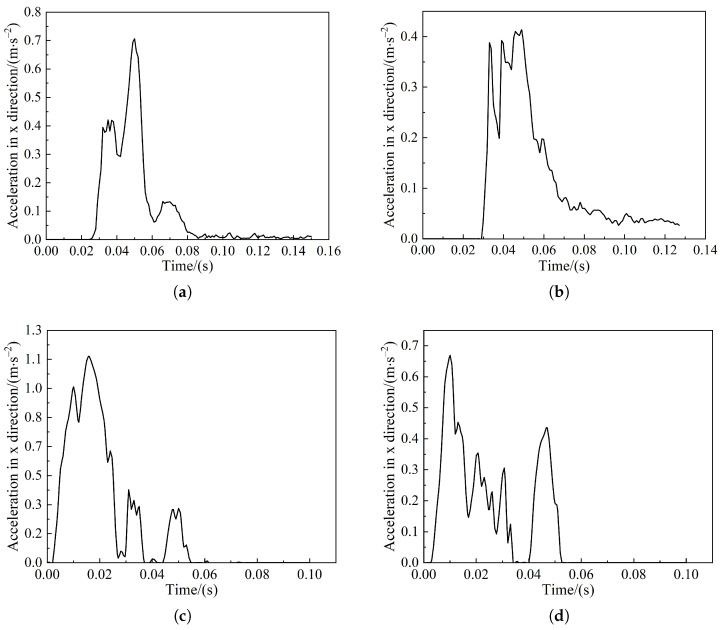
Acceleration curves of the train: (**a**) Pig head-on collision; (**b**) Pig offset collision; (**c**) Cow head-on collision; (**d**) Cow offset collision.

The acceleration curves of the train in frontal and offset collisions of the pig model are shown in Figure 23a,b, and the acceleration curves of the train in frontal and offset collisions of the cow model are shown in Figure 23c,d. It can be seen that the acceleration of the train from the frontal collision is higher than that from the offset collision, and the car body is especially obvious in the collision with the pig model. Regardless of the frontal collision or the offset collision, the car body acceleration generated by the collision with the cow model is greater. Therefore, there is a greater risk of train collisions with the cow. Nevertheless, in these collisions, the maximum train acceleration was generated in frontal collisions of the car body with cattle, which was 0.117 g, accounting for only 2.3% of the standard 5 g. Therefore, post-crash body acceleration satisfied the standards of EN15227.

### 5.2. Wheel Pair Lifting Volume

**Figure 24:**
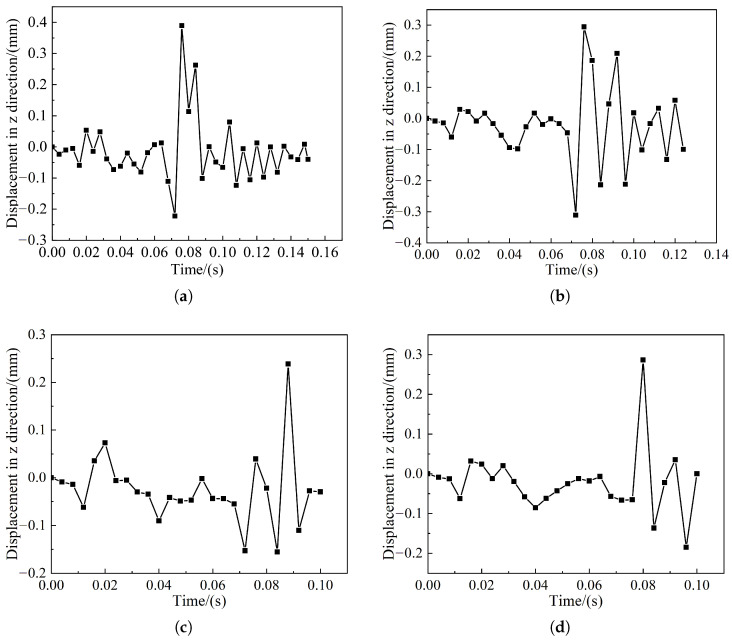
First round to lift volume change curve: (**a**) Pig head-on collision; (**b**) Pig offset collision; (**c**) Cow head-on collision; (**d**) Cow offset collision.

The first bogie of the head car of the train lifts the first wheel pair to the maximum height during the collision. Therefore, all the measured wheel pair lifts relate to the first wheel pair. Figure 24 shows the change curve of the first wheel pair lift of the first bogie. In Figure 24, the maximum lift of the wheel pair is between 0.2 mm and 0.4 mm, and the maximum value is 0.4 mm in the frontal collision with the pig. The nominal height of the locomotive wheel rim is 28 mm, and the EN15227 standard requires that the wheel pair lift should not exceed 75% of the nominal height of the wheel rim, which is 21 mm. Therefore, it is within the safe range and satisfies the requirements of EN15227 on the risk assessment of climbing vehicles.

### 5.3. Cowcatcher Deformed by Force

As can be seen from Figure 20, due to the height of the cow model, the collision first occurred with the coupler and anti-creeper, not the cowcatcher. The result of the collision shows that the scattered or destroyed tissues will collide with the cowcatcher. At this time, the force on the cowcatcher is small and not severe enough to cause damage to the cowcatcher; therefore, this paper only analyzes the performance of the cowcatcher when the train collides with the pig model.

**Figure 25:**
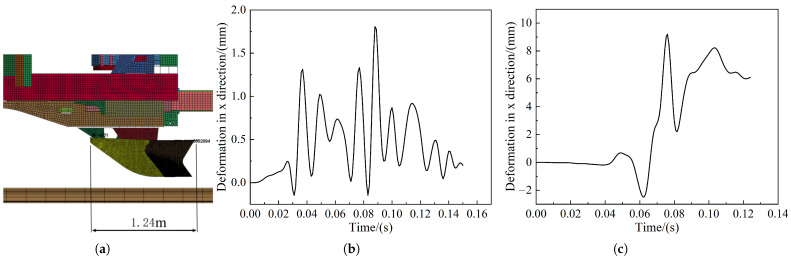
Deformation of the cowcatcher situation: (**a**) Schematic diagram of the location of the deformation test point of the cowcatcher; (**b**) deformation curve of pig frontal collision cowcatcher; (**c**) deformation curve of pig offset collision cowcatcher.

**Figure 26:**
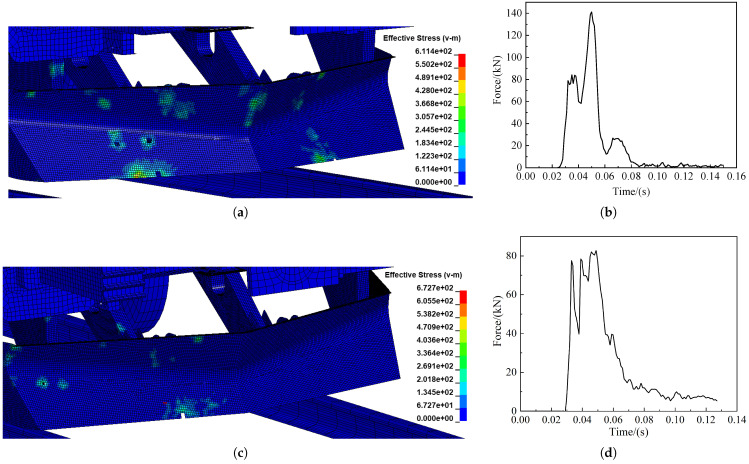
Changes in force on the cowcatcher: (**a**) Effective tress cloud diagram of the cowcatcher at 55 ms of collision; (**b**) Force curve of pig frontal collision cowcatcher; (**c**) Effective tress cloud diagram of the cowcatcher at 88 ms of collision; (**d**) Force curve of pig offset collision cowcatcher.

The distance between the two points was measured on a node taken at the head and tail of the excluder, respectively, as shown in Figure 25a. The deformation of the cowcatcher was determined using the longitudinal displacement difference of the node pair. Figure 25b shows the deformation curve of the cowcatcher in frontal collision with the pig; the maximum deformation is 1.81 mm, i.e., the maximum reduction is about 7.3 mm per 5 m in length, which is much smaller than the 50 mm allowed by the EN15227 standard. Figure 25c shows the deformation curve of the pig offset collision excluder, for which the maximum deformation is 9.2 mm, i.e., the maximum reduction for every 5 m in length is about 37.306 mm, which meets the standard requirements. Figure 26a,c shows the force cloud diagram of the cowcatcher, and Figure 25 shows the change of force on the cowcatcher. From Figure 26b,d, it can be found that the force on the cowcatcher reaches 140 kN at 55 ms of the collision during frontal collision, while the force on the offset collision excluder is only 80 kN, which is about 43% lower than that of the frontal collision. Both values are lower than the central static load on the cowcatcher delineated in the literature [21] 150 kN requirement.

### 5.4. Coupler and Anti-Creeper

As can be seen from Figure 20, due to the low height of the pig model, the objects directly collide with the cowcatcher, and the coupler and the anti-creeper are not involved in the collision; therefore, this section of the text only analyzes the force of the coupler and the anti-creeper when the collision occurs between the train and the cow model.

**Figure 27:**
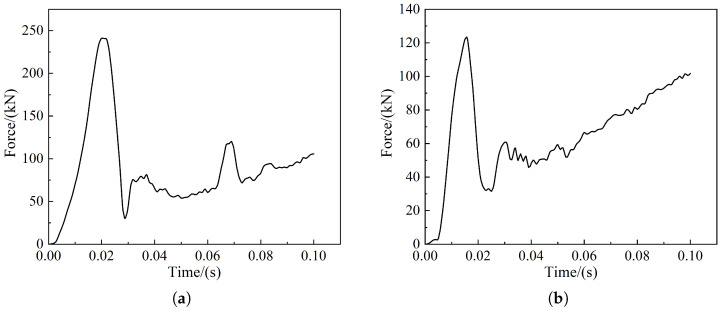
Coupler force curve: (**a**) Cow head-on collision; (**b**) Cow offset collision.

**Figure 28:**
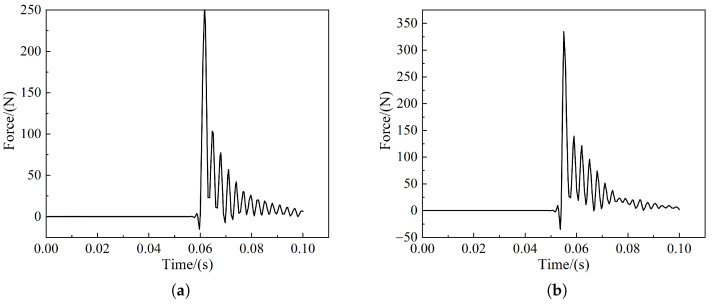
Anti-creeper force curveforce curve: (**a**) Cow head-on collision; (**b**) Cow offset collision.

From Figure 27 and Figure 28, it can be seen that when frontal collision occurs between the train and the cow, the force on the coupler is 242 kN and the maximum force on the anti-creeper is 230 N; however, when the offset collision occurs, the force on the coupler drops to 122 kN and the maximum force on the anti-creeper is raised to 334 N. Among them, the maximum force on the coupler is lower than the requirement of the maximum coupler force of 1000 kN for the train, and the coupler and the anti-creeper do not experience any force in the process of collision simulation; therefore, the couple and the anti-creeper did not suffer any deformation and damage. The normal operation of the train can be ensured after the collision.

## 6. Discussion and Conclusions

The three-dimensional finite element laminated model of a living organism proposed in this paper is different from the human model in the cab of a car or locomotive during a collision, and the main target of the living intruder model is the object of the collision, not the living intruder model itself. In contrast, previous studies have used dummy models to study the injuries caused by secondary collisions of passengers, etc. [19,25]. More attention has been focused on the damage caused to the model itself, ignoring or rarely considering the post-crash performance of the car. Due to the different research objectives, the solid modeling of the tissues in such studies is more elaborate than that of the organism model in this paper. Nevertheless, the validation method of the model in this paper is still based on the validation criteria of the refined model and against the actual experimental data.

In this paper, two organism models, of a pig and cow, were developed to simulate different species collisions, and frontal and offset collisions were performed for each animal body model. The results obtained at the end of the collisions are summarized in Table 9, where all values represent the maximum values generated during the collisions. As can be seen from the table, the maximum train acceleration generated by the cow model is significantly higher than that of the pig model in the case of frontal collision, while the maximum wheel pair lift is diametrically the opposite. The reason is that the cow model first makes contact with the coupler when the collision occurs, and the wheel pair lift is not very high because the coupler absorbs part of the energy generated by the collision, resulting in a relatively smooth collision process. Between different models, the maximum train acceleration in the offset collision was decreased. The maximum force on the cowcatcher in the offset collision of the pig model decreased, while the maximum deformation of the cowcatcher increased significantly. The reason for this is that the cowcatcher can only be counteracted by its structure when it is subjected to a unilateral force, while the frontal collision has a more uniform force, which will produce a larger deformation even if the force is not very large. In the offset collision of the cow model, the same contact with the coupler occurs first, but the coupler collides with the rear of the cow model and deviates from the center of gravity point; therefore, the maximum force on the coupler is reduced by about half compared with the frontal collision. In summary, all safety indicators of the train are related to the type of intrusion and the location of the collision. Nevertheless, all indicators are within the safety requirements.

Combining the four collision scenarios and the data in Table 9, we were able to draw conclusions about the safety of the train after the collision and speculate on the critical factors affecting the safety of the train. The results of the study showed the following.

(1) The filled 3D laminated model is close to the trend of experimental simulation results of the experimental literature results, which can ensure the accuracy of the model, while the model simplification reduces the modeling workload and improves the computational efficiency.
(2) In the case of 110 km/h and other collision positions, the collision risk factor between the train and heavier organisms is relatively high, and the risk arising from frontal collisions is generally greater than that of offset collisions; despite this, all the indicators such as the maximum acceleration of the train, the maximum lift of the wheel pairs, the reduction in the length of the cowcatcher discharge per 5 m of space, and the maximum central load borne by the cowcatcher discharge are lower than the EN15227 standard, the safety of the train is not affected, and the components can work reliably.

It is worth noting that our calculation method facilitates a more intuitive determination of the driving conditions after a train collision compared to the traditional calculation method, which is beneficial to ensure the safety of the train during its travel. However, we must admit that in the above work, we did not consider the case of a collision with other living intruders. Pigs and cows do not represent all possible intruding animals, and different animals are bound to cause different consequences, which will be one of the directions for future research. Secondly, the initial position of the collision is uncertain when the train collides with a live intruder, although it is considered that most animals will sprint laterally when frightened rather than running toward an open track, and even then there will be varied cases. In future research, we need to improve our calculation method by building multiple organism models and considering other collision locations.

## Figures and Tables

**Table 1:** Pig and cow outline size table.

Live Invader	Long (mm)	Wide (mm)	High (mm)	Weight (kg)
Pig	1201.3	404.2	697.8	120.13
Cow	2044	650	1320	600

**Table 2:** Elastic tissue material parameters.

Organization	Material Type	Density (kg/m${}^{3}$)	Young’s Modulus (GPa)	Poisson’s Ratio
Bone	Plasticity	2000	11.5	0.3
Skin	Flexibility	1000	0.035	0.42
Muscle	Flexibility	1200	0.0008	0.4

**Table 3:** Internal tissue material parameters.

Components	Density (t/mm${}^{2}$)	Material Properties	Material Parameters
Internal Organization	1.04	Viscoelastic	*G*${}_{0}$ = 0.528 MPa, *G∞* = 0.168 MPa, β = 35/s, *K* = 500 MPa

**Table 4:** Ligamentous tissue material parameters.

Components	Modulus of Elasticity (MPa)	Poisson’s Ratio	Cross-Sectional Area (mm${}^{2}$)
Ligaments	15	0.49	3.16

**Table 5:** Values of specific gravity of parts.

Organization Name	Specific Gravity
body	72.94%
Internal organs	7%
Fat	1.7%
Blood	1.11%
Other	17.25%

**Table 6:** Train material parameters.

Structure	Density(t/m${}^{3}$)	Elastic Model/(Gpa)	Poisson’s Ratio	Yield Strength/(Mpa)
Steering rack	5.725	205	0.3	345
Train body	7.9	70	0.3	250
Car Hook	3.545	200	0.3	350
cowcatcher	7.87	210	0.3	450

**Table 7:** Crashworthiness design categories of rail vehicles.

Category	Definition	Examples of Vehicle Types
C-I	Vehicles except urban vehicles and trams designed to operate on international, national and regional networks.	Locomotives, coaches and trains
C-II	Urban vehicles designed to operate only on a dedicated rail network, with no level crossings and no interface with road traffic.	Metro vehicles
C-III	Vehicles designed to operate on urban and/or regional networks, in track-sharing operation, and interfacing with road traffic.	Tram-trains, peri-urban trams
C-IV	Trams.	

**Table 8:** Selected collision scenarios.

Category	Collision Barrier	Crash Speed (km/h)
C-I	Small or low obstacles	110

**Table 9:** Crash simulation data summary table.

Intruder	State	Train Acceleration (g)	Wheel Pair Lift (mm)	Cowcatcher Deformation (mm)	Cowcatcher Force (kN)	Coupler Force (kN)	Anti-Creeper Force (N)
Pig	Head-on collision	0.067	0.4	1.81	140	Small or no	Small or no
	Offset collision	0.041	0.3	9.2	80	Small or no	Small or no
Cow	Head-on collision	0.117	0.24	Small or no	Small or no	242	230
	Offset collision	0.067	0.28	Small or no	Small or no	122	334

## Data Availability

Not applicable.

## Abbreviations

The following abbreviations are used in this manuscript:

CRRC	China Railway Rolling Stock Corporation
CT	Computer Tomography
MRI	Nuclear Magnetic Resonance Imaging
MDPI	Multidisciplinary Digital Publishing Institute
